# Are assortative mating and genital divergence driven by reinforcement?

**DOI:** 10.1002/evl3.85

**Published:** 2018-10-16

**Authors:** Johan Hollander, Mauricio Montaño‐Rendón, Giuseppe Bianco, Xi Yang, Anja M. Westram, Ludovic Duvaux, David G. Reid, Roger K. Butlin

**Affiliations:** ^1^ Department of Biology, Aquatic Ecology Lund University 223 62 Lund Sweden; ^2^ Department of Animal and Plant Sciences University of Sheffield S10 2TN Sheffield United Kingdom; ^3^ Current address: IST Austria Am Campus 1 3400 Klosterneuburg Austria; ^4^ Current address: UMR 1202 BIOGECO ‐ INRA/Université Bordeaux Site de Recherches Forêt Bois de Pierroton 69 route d'Arcachon 33612 CESTAS Cedex France; ^5^ Department of Life Sciences Natural History Museum SW7 5BD London United Kingdom; ^6^ Department of Marine Sciences University of Gothenburg 405 30 Gothenburg Sweden

**Keywords:** genitalia, gene‐flow, reinforcement, reproductive character displacement, speciation, Littorinidae, reproductive interference, assortative mating

## Abstract

The evolution of assortative mating is a key part of the speciation process. Stronger assortment, or greater divergence in mating traits, between species pairs with overlapping ranges is commonly observed, but possible causes of this pattern of reproductive character displacement are difficult to distinguish. We use a multidisciplinary approach to provide a rare example where it is possible to distinguish among hypotheses concerning the evolution of reproductive character displacement. We build on an earlier comparative analysis that illustrated a strong pattern of greater divergence in penis form between pairs of sister species with overlapping ranges than between allopatric sister‐species pairs, in a large clade of marine gastropods (Littorinidae). We investigate both assortative mating and divergence in male genitalia in one of the sister‐species pairs, discriminating among three contrasting processes each of which can generate a pattern of reproductive character displacement: reinforcement, reproductive interference and the Templeton effect. We demonstrate reproductive character displacement in assortative mating, but not in genital form between this pair of sister species and use demographic models to distinguish among the different processes. Our results support a model with no gene flow since secondary contact and thus favor reproductive interference as the cause of reproductive character displacement for mate choice, rather than reinforcement. High gene flow within species argues against the Templeton effect. Secondary contact appears to have had little impact on genital divergence.

**Impact summary**

How does assortative mating evolve during speciation? Our study provides a unique example where it is possible to distinguish among hypotheses explaining the evolution of reproductive character displacement. We test whether selection for reproductive isolation contributes to mating preference and genital divergence between sister species of mangrove snail. We find reproductive character displacement (greater differentiation in sympatry) for assortative mating but not for genital form. Assortative mating has evolved after cessation of gene flow, probably due to reproductive interference, so is not part of the speciation process. It may not depend on genital form, which could have diverged before secondary contact of the sister species. Crucially, a pattern suggesting the controversial process of reinforcement actually evolved after completion of speciation.

Reproductive character displacement (RCD), in which mating characteristics are more divergent between populations in areas of sympatry than in areas of allopatry, may suggest that the process of reinforcement has a role in speciation (Servedio and Noor 2003). Although the possibility of reinforcement is now widely accepted, its role in speciation remains controversial. Reinforcement is here defined as the evolution of enhanced reproductive isolation in response to selection against hybrids or recombinant genotypes (but see Butlin and Smadja 2018 for discussion of this definition). One setting in which reinforcement may occur is upon secondary contact of two taxa that have previously diverged in allopatry and between which there is partial reproductive isolation; in this context natural selection against hybrids may favor the evolution of stronger prezygotic isolation in the contact zone, hence permitting geographic overlap and generating the pattern of RCD (Coyne and Orr 2004). It is, however, well known that other processes can produce this same signal (Noor 1999). Taxa that are already fully reproductively isolated may nevertheless undergo selection for divergence in mating traits due to reproductive interference, leading to RCD (Butlin 1987a,b; Butlin and Ritchie 2009). Furthermore, Templeton (Templeton 1981) argued that the same geographic pattern of RCD can be generated when previously allopatric taxa come into secondary contact, if populations within each species vary in mating traits and sympatry is only possible between pairs of populations that are sufficiently differentiated to prevent interbreeding (Paterson 1978; Templeton 1981; Coyne and Orr 1989, 2004); this phenomenon is known as the ‘Templeton effect’ or ‘differential fusion’. In addition, character displacement may be driven by ecological, rather than reproductive effects.

Although reinforcement, reproductive interference, and the Templeton effect can all create a pattern of RCD, i.e., greater trait divergence and stronger assortative mating in sympatry than in allopatry, they require different conditions and reflect different histories. Therefore, they can be distinguished. A signature of past gene flow that is now reduced or absent supports reinforcement. Reproductive interference requires evidence for wasteful mating interactions in sympatry, perhaps with the production of early‐generation hybrids (F1 hybrids), but no history of gene flow. The Templeton effect requires mating discrimination to evolve among partially isolated populations within each incipient species while they are allopatric to enable some pairs of populations, but not others, to coexist without fusion after secondary contact. This predicts that mating trait divergence and assortative mating between species will fall within the range of divergence or assortment observed between populations within species. The Templeton effect may be associated with some reduction in gene flow between conspecific populations that differ in mating traits. Gene flow between species after secondary contact is expected to be low or absent.

Interpretation of an observed pattern of RCD requires a distinction among these three possibilities and, if possible, to exclude ecological character displacement. Some comparative studies have revealed patterns that appear to be specific to reinforcement (Coyne and Orr 1989, 1997; Yukilevich 2012), but direct analysis of individual species pairs may be the only way to make the distinction in other taxa (Hollander et al. 2013). Few case studies exist that provide enough information to distinguish among the three possibilities, and they are strongly biased toward providing evidence for reinforcement (Pfennig 2003; Lemmon and Lemmon 2010; Bimova et al. 2011; Hopkins and Rausher 2011). This may be because reinforcement is a common component of speciation, but it may also reflect the choice of study systems or a reporting bias.

One class of traits that may show RCD is genital form. Genital form is hugely variable among animal species, and evolutionary biologists are still seeking to explain its rapid divergent evolution (Eberhard 2004; Eberhard 2010; Simmons 2014). Male genital form is often easily observed and is an essential character for identification of species in many animal taxa. Female genital variation is more cryptic (but see Anderson and Langerhans 2015) and yet probably underlies key interactions during mating (Eberhard 2010). Genital divergence has been studied most intensively in insects and spiders and several hypotheses have been proposed to explain variation in male intromittent genitalia, such as cryptic female choice (Thornhill 1983; Eberhard 1985; Arnqvist and Rowe 2005), male–male competition (Seehausen and Schluter 2004), manipulation of sperm competition (Waage 1979; Cordero‐Rivera 2017), or sexually antagonistic coevolution (Arnqvist and Rowe 2005). Eberhard (Eberhard 2004) conducted a large‐scale comparative analysis within these taxa, but did not find support for either sexually antagonistic coevolution or cryptic female choice (Eberhard 2010) as general explanations for genital divergence.

Genital divergence that has evolved by any of these mechanisms can contribute to reproductive isolation and hence to speciation (Arnqvist 1998), although as an incidental consequence of divergence. In contrast, early studies viewed genitalia as a ‘lock and key’ mechanism, whose purpose was to prevent heterospecific insemination (Eberhard 1985). This mechanism implies that genital divergence can be driven by reinforcement or reproductive interference (Butlin and Ritchie 2013). However, the attempts to test the possibility of genital divergence due to reinforcement or reproductive interference have been few (Kameda et al. 2009; Kuntner et al. 2009; Hollander et al. 2013).

Comparative analysis of genital form in a large clade of dioecious marine snails (Caenogastropoda: Littorininae) revealed a strong pattern of greater difference in penis form between sister‐species pairs with overlapping ranges than between allopatric pairs (Hollander et al. 2013). Variation in genital morphology is a striking feature in littorinid gastropods, widely recognized in the taxonomic literature (Reid 1986, 1996). Therefore, littorinids provide a very promising model system to address questions concerning the role of genital form in the evolution of reproductive isolation and the role of reinforcement in speciation. Forty genetically identified sister‐species pairs with well‐characterized distributions were available (Reid et al. 2012) for analysis and the pattern was robust to controlling for species age. These results (Hollander et al. 2013) suggest that genital form in the Littorinidae may have diverged as a result of selection against hybrids, representing a putative signal for reinforcement that demands further analysis to exclude alternative possibilities. In particular, the comparative analysis raises three questions: Is genital divergence associated with assortative mating? Is there a geographical pattern of reproductive character displacement within species? What is the evolutionary origin of reproductive character displacement?

Here, we test for the geographical pattern of reproductive character displacement in mate choice and genital form in one sister‐species pair of littorinids, *Littoraria cingulata* and *L. filosa*, and distinguish between three competing explanations for this pattern. These snails live permanently above the water level on mangrove trees, but reproduce by spawning pelagic larvae with wide dispersal (Reid 1986). *Littoraria cingulata* is endemic to Western Australia, while *L. filosa* is found mainly in Northern and Eastern Australia. There is a region of geographic overlap around Broome, in the northern part of Western Australia. In both allopatry and sympatry, the species occupy slightly different but overlapping horizontal zones within the mangrove forest, but often reside at the same supratidal levels and on the same trees (Reid 1985). This pattern argues against RCD as a side effect of ecological character displacement. They are known to differ in penial form, although not diagnostically so (Reid 1986, 2001). Interspecific mating is frequent, but hybrids have not previously been recognized (Reid 1986).

We demonstrate strong RCD for mate choice but not for genital form. Demographic reconstruction rejects models with gene flow since secondary contact and yet we report an F1 hybrid individual from the field. Overall, our results strongly support reproductive interference as the cause of character displacement for mate choice, rather than either reinforcement or the Templeton effect, while genital divergence preceded secondary contact and its cause remains unknown.

## Results

### REPRODUCTIVE CHARACTER DISPLACEMENT

For each combination of sex, population, and species, 60 mating trials were conducted resulting in a total of 960 trials, of which 940 provided data for analysis. Mounting occurred in 332 trials and penis insertion was observed for 67.5% of mountings. Assortative mating was significant in sympatry (*I*_PSI_ = 0.55 ± 0.08, *t* = 7.25, *n* = 119, *P* << 0.001), but not in allopatry (*I*_PSI_ = 0.13 ± 0.08, *t* = 1.88, *n* = 215, *P* = 0.061) (Table 1). These levels of assortment were significantly different, as judged by the male species × female species × location interaction in the GLM (χ^2^ = 5.699, *df* = 1, *P* = 0.017), demonstrating RCD for mate choice.

**Table 1:** Mating trial outcomes. The body of the table gives the counts of trials in which one or more mountings were observed in the 2‐h observation period, out of a total number of trials (in parentheses)

Sympatric		Male	
	*Littoraria cingulata*		*Littoraria filosa*
**Female**			
*L. cingulata*	54 (117)		4 (115)
*L. filosa*	28 (117)		33 (119)
			
Allopatric		Male	
	*Littoraria cingulata*		*Littoraria filosa*
**Female**			
*L. cingulata*	38 (118)		40 (117)
*L. filosa*	49 (118)		86 (119)

**Figure 1:**
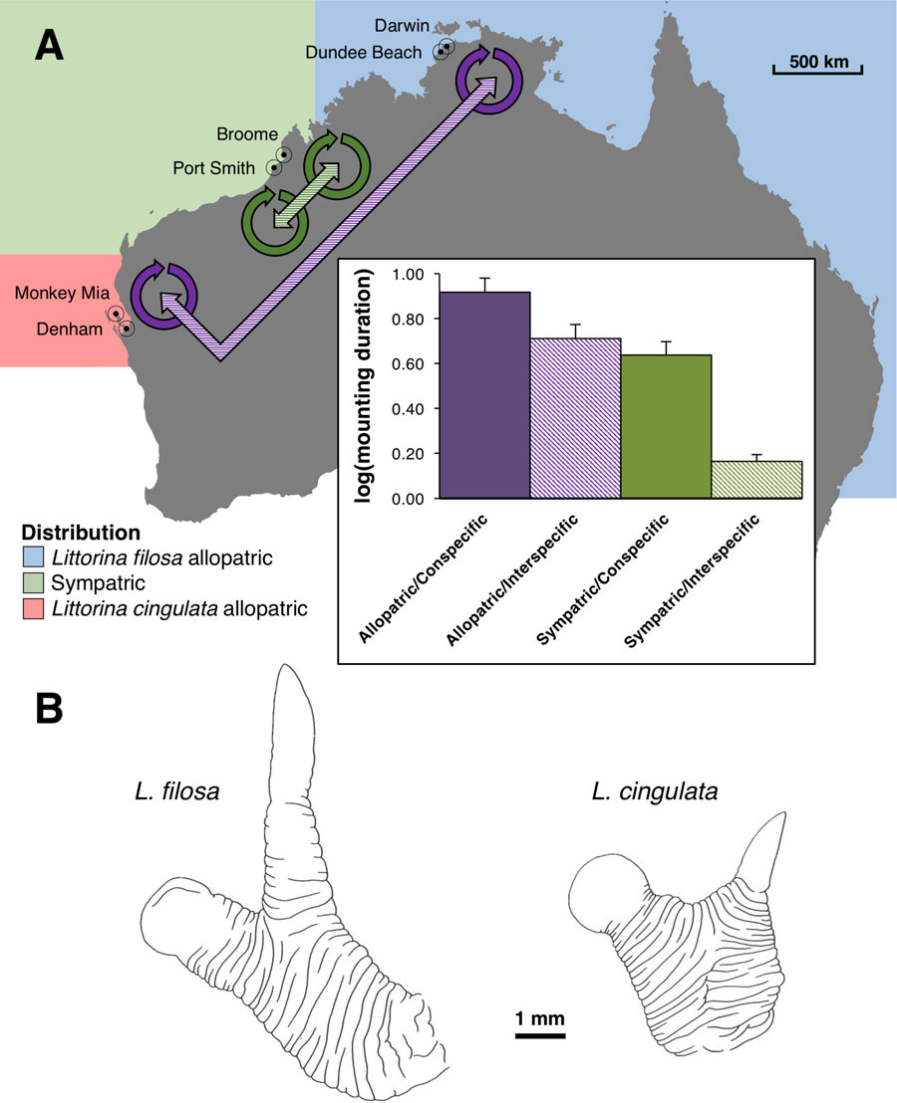
(A) Map of the sampling sites from the north and west coasts of Australia. Coloured regions describe the snail species distribution areas. Colours correspond with Fig. 2. Arrows designate combinations between pairs of populations for which mating trials where conducted, while the bar plot shows the mating trials outcomes in terms of mounting duration (minutes). Error bars represent SE. (B) Camera lucida drawings of the penes of *L. filosa* and *L. cingulata*, showing the elongated filament of the penis together with the penial glandular disc attached to the wrinkled base (after Reid 1986).

Mounting durations varied from 1 to 700 min and the probability of finding sperm in the female bursa at the end of a mating trial was strongly dependent on the average duration of matings during the trial (*b* = 0.59 ± 0.13, χ^2^ = 23.26, *df* = 1, *P* << 0.001; logit scale). Since duration apparently influenced the success of mountings, we tested for an effect of location and found a highly significant male species × female species × location interaction (χ^2^ = 18.89, *df* = 1, *P* = 0.00022), driven mainly by short interspecific matings in the sympatric, but not in the allopatric combinations (Fig. 1A).

Penial form differed between species, as expected, but the difference was not greater in sympatry than in allopatry (distances between the centroids in multivariate shape space: allopatry 0.733, sympatry 0.816, *P* = 0.215 by permutation; Fig. S3). The trajectories did differ in direction (*P* = 0.005), explaining an overall species × location interaction (*F*_1,780_ = 10.67, *P* = 0.005). Thus, the overall prediction of RCD was not supported but the change in vector suggested that some aspects of penis form did differ more in sympatry than in allopatry. Analyzing individual variables, we found significant species × location interactions (*P* < 0.05 after Bonferroni correction) for six traits (B, G, H, I, J, K). The clearest pattern of character displacement was for trait G, the width of the glandular side branch of the penis (Table S2).

PC1 (46% of variation) showed strong differentiation between species. When this variable was added to the GLM for mating duration, there was a significant interaction between male species and PC1 (*F*_1,227_ = 6.61, *P* = 0.022) with the slope less negative for *L. filosa* males than for *L. cingulata* males, as might be expected because *L. filosa* had the higher mean score. However, there was no significant interaction with location and so no evidence that penis form contributed to the pattern of RCD in mating duration.

### GENE FLOW

**Figure 2:**
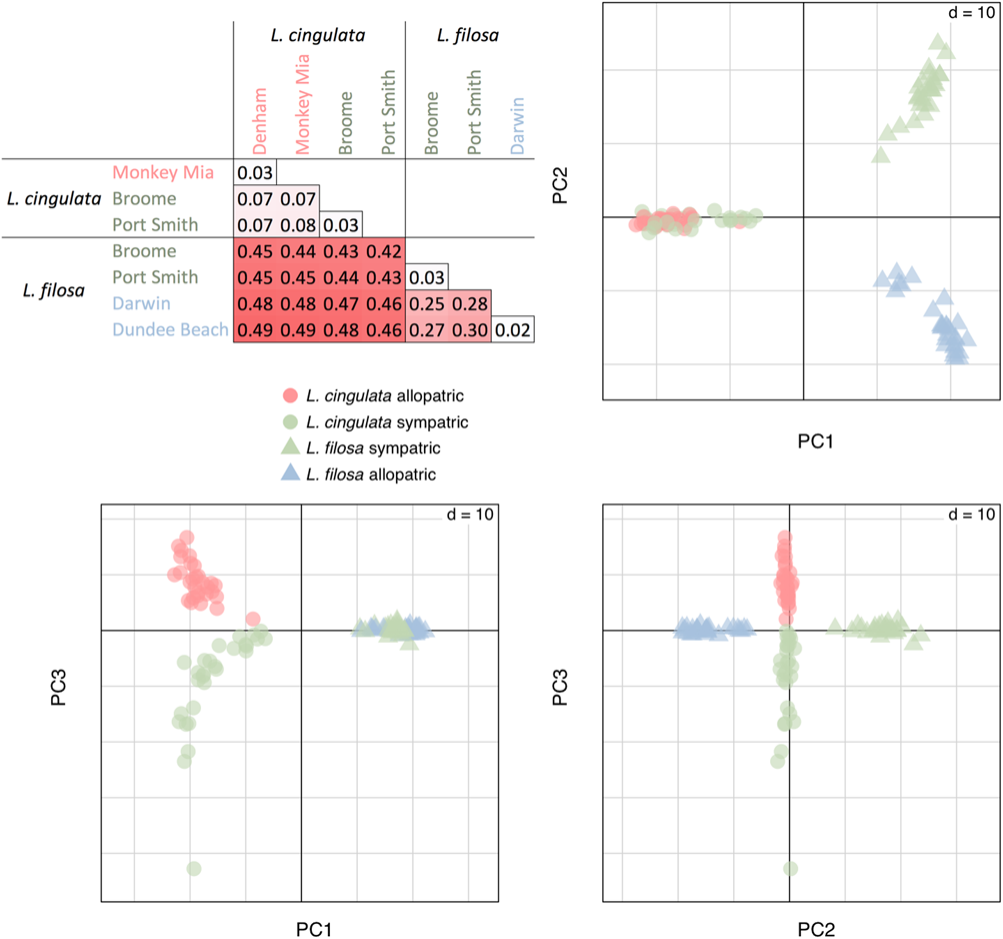
The top left panel gives Nei's estimator of pairwise F_ST_ values; locality names in red and blue represent allopatric populations of *L. cingulata* and *L. filosa*, respectively; those in green represent sympatric populations of both species. The following panels show principal component scores from the first three axes of an analysis based on 1878 loci in 113 individuals. Circles (allopatric) and triangles (sympatric). Each point represents a single individual.

Genetic data were obtained for 113 individuals, and 1920 SNPs (one per tag) passed filters for the PCA and F_ST_ analysis. We found no evidence for hybrids in the set of 113 individuals as the species formed separate clusters in the PCA (Fig. 2). Populations of the same species were genetically very similar within regions. Allopatric and sympatric regions were more differentiated for *L. filosa* than for *L. cingulata*, and sympatric *L. cingulata* were very slightly more similar to *L. filosa* than allopatric *L. cingulata* were. This could be a signal of gene flow but, in the ABC analysis, the historical demographic model with no migration was strongly preferred over the next best model (posterior probability for No migration: PP = 0.83, Constant migration: PP = 0.02, Recent migration: PP = 0.12, Ancient migration: PP = 0.03). Analysis of pseudo‐observed datasets showed good power to distinguish the models (Fig. S4) and the No migration model was the only model capable of explaining the observed data adequately (Fig. S5). The parameter estimates for the preferred model (Table S4 and Fig. S6) suggest that the species separated approximately 500,000 years ago with regions within *L. filosa* separated for longer (∼250,000 years) than regions within *L. cingulata* (∼125,000 years), but with similar levels of intraspecific gene flow between regions (∼0.01 migrants per generation). Population sizes were estimated to be large in the present (∼10^6^ individuals) but smaller in the past (∼10^5^ individuals), consistent with contraction during Pleistocene climatic cycles.

The putative hybrid individual, identified in the field on the basis of intermediate shell characteristics, was heterozygous for all 10 putatively‐diagnostic SNPs for which it was genotyped (Table S5). Nine of these 10 SNPs were homozygous, for species‐specific alleles, in all individuals identified as *L. filosa* or *L. cingulata* from the Broome sympatric sample site (*n* = 3–7), with one *L. cingulata* heterozygous at locus 458736. This result clearly identifies the putative hybrid as an F1.

## Discussion

Divergence in genital form, even between closely related species (Eberhard 2010), provides valuable traits for taxonomists and implies rapid evolution. However, only a few studies have tried to distinguish among forms of selection driving genital form. A comparative analysis in the Littorininae (Hollander et al. 2013) revealed a pattern of greater divergence in penis form between sister‐species pairs with overlapping ranges than between pairs with allopatric distributions. This study raised several questions that we have addressed here. Is genital divergence associated with assortative mating? Is the comparative pattern associated with a geographical pattern of RCD? If so, is this due to reinforcement, reproductive interference between species, or the Templeton effect? This last question is important for the study of reinforcement in general (Butlin 1987b; Coyne and Orr 2004), but it is difficult because it requires inferences about historical gene flow and patterns of variation among populations in reproductive traits.

*Littoraria cingulata* and *L. filosa* were one of the sister‐species pairs included in the comparative analysis by Hollander et al. (2013), characterized by extensive range overlap, low penial similarity (0.918, compared with allopatric species pairs with mean similarity 0.949 ± 0.011), and genetic distance 0.036 (estimated divergence time in the range 1–4.5 million years ago (Reid et al. 2012)). Our analysis confirms the difference in penial form, and shows some differences in form between populations in the sympatric region and those in the allopatric regions (Fig. S3). However, the sympatric–allopatric differences were overall in the same direction for the two species and similar in magnitude such that the difference between species was maintained and there was no overall pattern of RCD. Penial form was weakly associated with mating duration, suggesting the possibility of selection on shape due to interactions between the male and female during copulation (as suggested by Hollander et al. 2013), but this did not help to explain the strong signal of RCD in mating duration. Together, these results suggest that neither reinforcement nor reproductive interference has contributed to penial form divergence between *L. cingulata* and *L. filosa*, unless gene flow has allowed divergence that initially evolved in sympatry to spread throughout the species ranges. A species‐level, rather than population‐level, version of the Templeton effect remains viable. The comparative pattern of greater penial divergence in species with overlapping range (Hollander et al. 2013) could then be explained if range overlap was facilitated by divergence in penial form between species, because it reduced costly hybridization via an effect on assortative mating. However, some other factor would be required to explain the lack of complete range overlap. This explanation seems unlikely, given the strong RCD for mating duration that is independent of penis form.

Assortative mating, judged by mounting success, was significant for sympatric but not for allopatric populations. An even stronger pattern of RCD was seen for mating duration. During copulation, sperms are transferred first to the female's bursa but then quickly transferred to the seminal receptacle for storage (Reid 1986), so sperm in the bursa indicate recent mating. Since the females we collected from the field were probably not virgin, some may have had sperm in the bursa from matings that occurred before collection. Nevertheless, the strong correlation that we observed between mounting duration and the presence of sperm in the bursa demonstrates that longer mounts are more likely to result in sperm transfer. Thus, the combination of lower mounting probability and shorter mounting duration for interspecific pairs from sympatric populations represents a much stronger barrier to hybridization in sympatry than in allopatry. The patterns were consistent across the two populations that we sampled in each region, however additional populations would strengthen the inference that co‐existence and divergence are causally connected.

Is the stronger barrier in sympatry a result of reinforcement? Our historical reconstruction assumed divergence of *L. cingulata* and *L. filosa* during a period of allopatry and tested the occurrence of gene flow during the current secondary contact. It showed reduced population size before the present secondary contact. Histories like this have been suggested to be typical for the radiation of littorinids (Reid et al. 2010, 2012). However, our model comparisons strongly support the No‐migration model in which reproductive isolation was complete at the time of secondary contact. The next‐best model was the Recent migration model, but there was good power to discriminate models and the observed summary statistics were consistent with the No migration model and not with the Recent migration model. Reinforcement would be a reasonable interpretation of the observed pattern of reproductive character displacement only under the Constant migration or Ancient migration models and so can be rejected.

Despite evidence that gene flow has not occurred since secondary contact, we found one F1 hybrid. These observations are consistent if F1 or backcross fitness is very low, so that occasional hybridization occurs but does not lead to gene exchange. The discovery of this hybrid individual, combined with the observation of interspecific mounting and insemination in our mating experiments, as well as frequent mountings in the field, demonstrates the potential for reproductive interference to have generated the observed pattern of reproductive character displacement, because costly interspecific matings do occur in sympatry.

The Templeton hypothesis is difficult to exclude. However, our demographic models suggest gene flow between sampled regions, within each species, even though they are separated by about 1500 km. This is consistent with a planktonic period thought to be in the region of 3 to 10 weeks, resulting in dispersal over hundreds of kilometres (Reid 1986; Reid et al. 2010). Many intervening populations are known to occur (Reid 1986). Therefore, it is unlikely that there was any opportunity for isolated populations to diverge in mating traits during the period when the species were allopatric, unless the mangrove habitat was severely restricted by climatic or sea‐level fluctuation during Pleistocene glacial cycles, or strong selection overcame gene flow. Noor (Noor 1999) also suggested sexual selection or ecological character displacement as alternative explanations for RCD. However, we agree with his conclusion that sexual selection is not really a distinct explanation since it is likely to be initiated by low fitness of hybrids (reinforcement) or costly interspecific interactions (reproductive interference). *Littoraria filosa* and *L. cingulata* each occupy the same habitats in both sympatric and allopatric locations (*L. filosa* is always at a higher level, on average, than *L. cingulate*; 35). Neither assortative mating, given contact, nor penial form are likely to be subject to nonreproductive selection pressures. Therefore, confounding effects of ecological character displacement are unlikely in this case. Given the evidence for costly interspecific interactions, reproductive interference following secondary contact is the most parsimonious explanation for the pattern of RCD and is consistent with all aspects of our data.

We provide a rare case study, distinguishing reproductive interference (Butlin and Ritchie 2013) from other causes of the stronger barriers to hybridization in sympatry than in allopatry. RCD is a common pattern (Coyne and Orr 2004). Much attention has focused on asking how often this pattern is due to reinforcement: for example, Yukilevich (Yukilevich 2012) concluded that reinforcement has enhanced reproductive isolation in 60–83% of sympatric *Drosophila* species. He also presented evidence against the Templeton effect, but did not consider the possible impact of reproductive interference, which might be the underlying cause for some cases that he assigned to reinforcement. The extent of natural hybridization and gene flow is not known for many of the species pairs in his analysis but would help to make this distinction. The effects of sympatry and the effects of gene flow cannot easily be separated in such comparative analyses (Nosil 2013), especially because production of viable hybrid offspring under laboratory conditions (cf. Coyne and Orr 1989, 1997) cannot be used to infer gene flow in the field. As emphasized previously by Noor (1999) and Butlin and Ritchie (2013), it is only when these various processes are adequately distinguished that it will be possible to assess their impacts on the origin of species.

## Methods and Material

### SAMPLING

Individuals of *L. cingulata* and *L. filosa* were collected from allopatric and sympatric sites in Australia in November 2013 (Table S1; Fig. 1A). Field identifications were based on diagnostic shell traits (*L. filosa* has a thinner, usually more finely ribbed, color polymorphic shell; Reid 1986) and were confirmed using genetic markers (see below). At one site (Monkey Mia), *L. cingulata* was collected from an atypical habitat under loose rocks on the shore.

Allopatric sites were hundreds of kilometers from the nearest known populations of the sister species, whereas the two species occurred syntopically at sympatric sites. Sites were selected based on previous sampling (Reid 1985, 1986). Two replicate localities separated by tens of kilometers were sampled for each species in each region (allopatric and sympatric). For each of these eight samples, mantle tissue was preserved in 100% ethanol from 30 females. Sixty males and 60 females per site were used for mating trials and penes from these males were preserved in 100% ethanol. Voucher specimens are preserved at the Natural History Museum (London).

### TEST FOR CHARACTER DISPLACEMENT IN MATE CHOICE

No‐choice mating trials were conducted among the following combinations: (i) allopatric conspecifics, (ii) allopatric heterospecifics, (iii) sympatric conspecifics, and (iv) sympatric heterospecifics, in both directions between males and females and always involving males and females from different sites, to avoid confounding assortment by site with assortment by species. To achieve all combinations, some individuals had to be transported between sites but we detected no mortality nor reduction in activity following transport.

Sex was determined by the presence or absence of the conspicuous penis in individuals of adult size. Mating trials were conducted in transparent plastic spheres (Ø 8 cm), with males identified by a mark on the shell. Trials were performed at ambient temperature under artificial illumination and snails were lightly sprayed with water at the start, to initiate activity. Two experimenters performed trials in batches of 20, three batches before noon and three after noon. Each trial lasted 2 h unless a pair was “in copula,” in which case the trial was extended until the mating finished. Individual snails were only used once. Batch order was balanced to minimize confounding of time since collection, time of day, or environment effects with population effects. Individual snails were chosen at random. We recorded the size (maximum shell length: *L. cingulata* males 10.5–24.0 mm, females 10.5–25.2 mm; *L. filosa* males 12.4–29.0 mm, females 14.6–29.8 mm) of the male and the female, and the time at contact, mounting, dismounting, penis insertion, and penis withdrawal. After a batch of mating trials, all mated females were anesthetized and dissected and the presence of sperm in the copulatory bursa was recorded. Subsequently, we relaxed and anesthetized all males alive in magnesium chloride (isotonic with seawater, 1:13 v/v dilution), for 30 min before the snails were killed with boiling water, to fix them in an extended position (Reid 1996). The penes were removed and preserved in 70% ethanol for morphometric analysis.

We used the program Jmating (Carvajal‐Rodriguez and Rolan‐Alvarez 2006) to obtain estimates of the isolation index, I_PSI_, in allopatry and in sympatry, based on the numbers of trials with and without mounting. I_PSI_ is a joint isolation index that is not affected by sexual selection. We also analyzed mounting as a binary response variable and mount duration (log‐transformed) as a continuous variable, using linear models with binomial or Normal error, respectively. We included as terms of interest: male species, female species, and location (allopatric vs sympatric) and their interactions, and potential confounding variables identified in preliminary analyses: researcher and starting time (morning vs afternoon) for mounting probability; and male size, female size, size difference, and female maturity (categorized after the mating trial by appearance of gonad and presence of spawned embryos in mantle cavity as immature, maturing, mature, and spent (Reid 1986)) for mating duration. Penis insertion was not seen for all mountings, but restricting mounting to cases where it was observed did not alter conclusions. Since sperm are stored in the bursa for only hours or a few days after insemination, before being transferred to the seminal receptacle (Reid 1986), a full bursa indicates recent successful copulation. We therefore tested the biological significance of longer mounting duration by asking whether it increased the probability of finding sperm in the bursa at the end of a trial, using logistic regression.

### TEST FOR CHARACTER DISPLACEMENT IN GENITAL TRAITS

Outlines of penes (Fig. 1B) from all snails included in the mating experiment were drawn using a stereo‐microscope with camera lucida attachment, with a scale bar, and then scanned in 8‐bit greyscale at a resolution of 600 dpi. The scanned image was imported into ImageJ version 1.49 (Abràmoff et al. 2004) and, using a custom macro, first converted to a binary image and then transformed using a series of morphological filters and distance maps (Burger and Burge 2008) to extract the key‐points from which 11 features were measured (Fig. S1, see Supplementary Methods: One feature was dropped because it could not be measured in all specimens and the first feature, overall length, was used to adjust the remaining nine measurements to the mean length, using linear regression).

The length‐adjusted penis measurements were analyzed using the ‘trajectory.analysis’ function in the *geomorph* package in R (version 3.0.4 (Adams and Otárola‐Castillo 2013; Adams et al. 2016)). We compared the trajectory in multivariate shape space from mean *L. cingulata* penis form to the mean *L. filosa* form in allopatric populations with the trajectory in sympatric populations, predicting that RCD would make the path distance for the latter greater, potentially also changing its direction. A principal component analysis (PCA) was performed within the trajectory analysis and we used scores on PC1, which contributed most to separating the species, in analyses of the impact of penis form on mating‐trial outcomes.

### TEST FOR RECENT GENE FLOW

DNA was extracted from 120 individuals (15 females from each of the four sites per species (Fig. 1A), not used in the mating experiment), using a modified version of the protocol of Wilding et al. (2001). These individuals were genotyped with a reduced‐representation sequencing approach. Beijing Genomics Institute (Hong Kong) prepared and sequenced DNA libraries and called SNPs using a protocol based on that of Andolfatto et al. (2011) and digestion with the *Ape*KI restriction enzyme. After pool amplification of the adapter‐ligated DNA fragments, these were size‐selected within a range of 300–600 bp, ensuring DNA insert length was 200–500 bp, considering that the length of the adapters was 100 bp. Paired‐end 90 bp sequencing was performed using an Illumina HiSeq system. After de‐multiplexing, adapter removal, and quality trimming, reads were assembled, allowing up to four mismatches, and SNPs were called. SNPs with fewer than five reads were removed and more than one read of each allele was required to call heterozygotes. For initial population‐genetic analysis, we used only the first SNP in each tag, thus avoiding treating SNPs in the same tag as independent from each other. The R package *adegenet* (version 1.4‐2 (Jombart and Ahmed 2011)) was used to compute PCA and pairwise F_ST_ between samples. SNPs that departed from Hardy–Weinberg expectations with *P* < 0.01 in any one of the four regions were excluded and only loci genotyped in >80% of individuals were retained for this analysis.

Given strong biogeographic evidence for divergence of the species in allopatry (Reid et al. 2010), we tested for gene flow following secondary contact by fitting four possible demographic models (No migration, Constant migration [since contact], Recent migration, Ancient migration; Fig. S2) to the genetic data using an approximate Bayesian computation (ABC) approach (Csilléry et al. 2010). For each species, sites within regions were merged since population genetic analyses revealed no evidence of differentiation at this level. For this analysis, we used tag sequences, rather than SNPs. Only one allele for each individual and tag was retained because of the uncertainty associated with genotype calling, and only tags with at least five individuals typed in each region were retained. All biallelic SNP positions were retained. We reduced the data set to five randomly chosen individuals per region, for ease of simulation. Monomorphic tags were then excluded, leaving 29,623 polymorphic 82 bp tags for analysis. Details of the ABC methods are provided in Supplementary Methods.

### PUTATIVE INTERSPECIFIC HYBRID

During sampling at Broome, a putative hybrid between *L. cingulata* and *L. filosa* was identified by DR, based on shell traits. This individual, along with seven reference individuals of each species, was genotyped for 10 putatively‐diagnostic SNPs identified from the reduced representation tags (Table S5, see Supplementary Methods).

Associate Editor: Z. Gompert

## Supporting information


**Figure S1**. Feature extraction from penis drawings (see *Supplementary Methods* for details).

**Figure S2**. Demographic models investigated in this study.

**Figure S3**. Trajectory analysis of penis form.

**Figure S4**. Posterior probabilities of models NM and RM over 100 rounds of leave‐one‐out cross‐validation analysis.

**Figure S5**. Principal component analysis of datasets simulated under models NM and RM (1e6 datasets under each model) using the *a priori* simulated summary statistics.

**Figure S6**. Posterior distributions of the NM model parameters.

**Table S1**. Collection sites of *Littoraria cingulata* and *L. filosa*.
**Table S2**. Analysis of individual penis traits (after size correction).
**Table S3**. Uniform prior distributions [low bound – high bound], after having ensured that priors included the posteriors.
**Table S4**. Parameter estimation under the NM model, chosen for having received the highest posterior probability.
**Table S5**. Primer sequences for 10 putatively diagnostic SNPs.
