# Supplementary figures and images for: Are assortative mating and genital divergence driven by reinforcement?
Source: Evol Lett. 2018 Oct 16;2(6):557–66. doi: 10.1002/evl3.85 (PMC6292706; doi:10.1002/evl3.85)

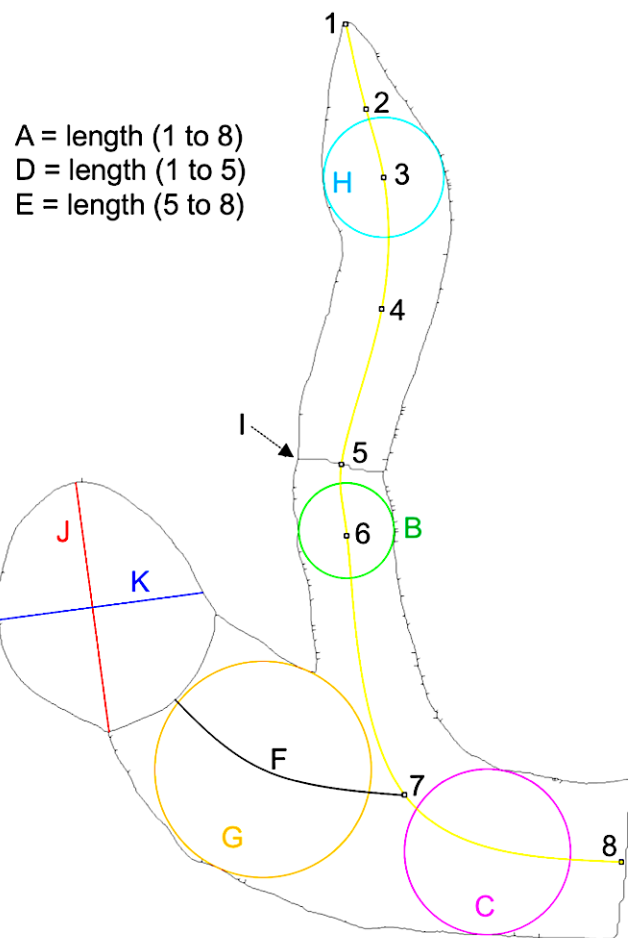

A = length (1 to 8)  
D = length (1 to 5)  
E = length (5 to 8)

Supplement: Supplementary file 1 — Figure S1. Feature extraction from penis drawings (see Supplementary Methods for details). [file EVL3-2-557-s001.pdf]

## Two Dimensional View of Phenotypic Trajectories

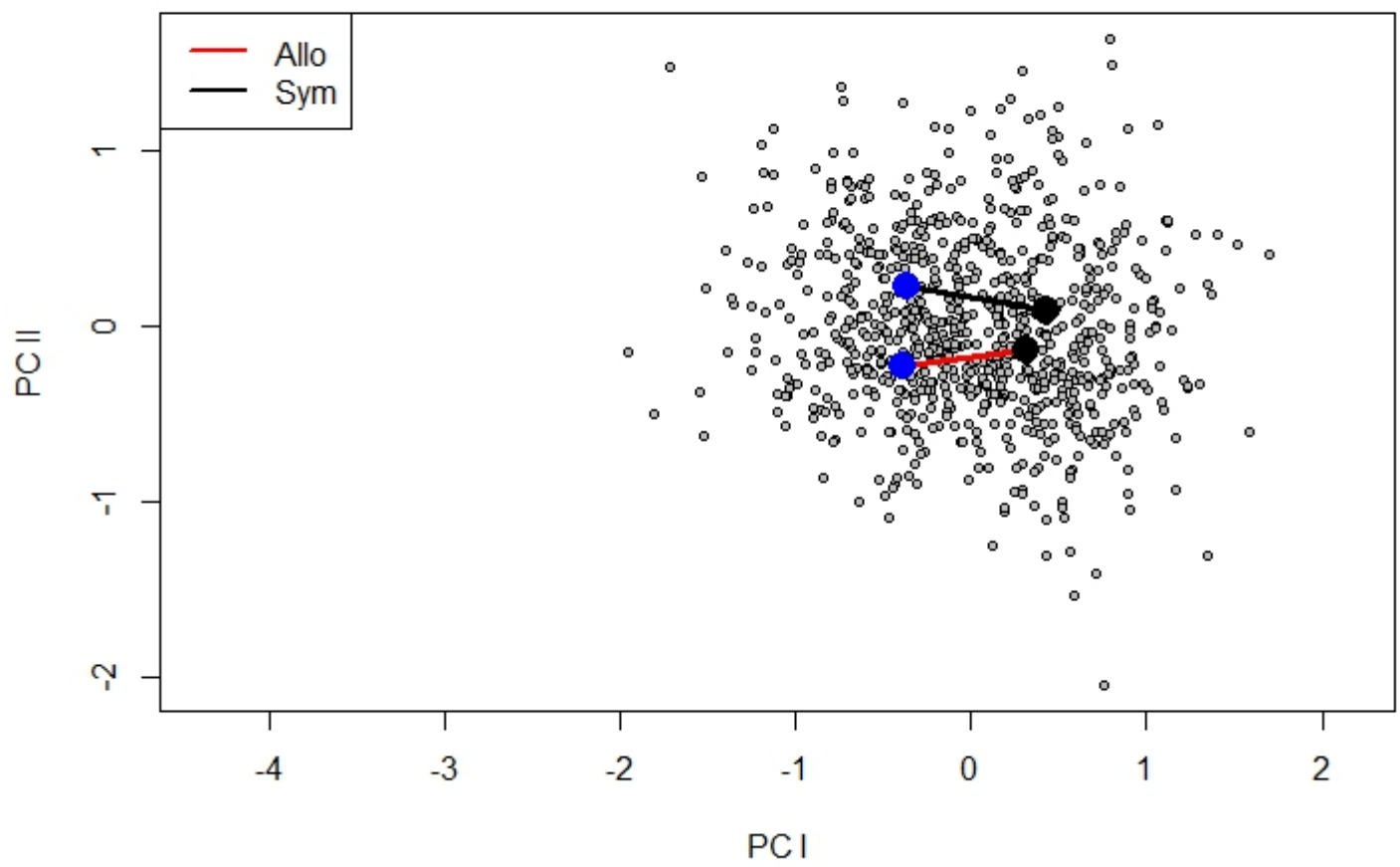

Supplement: Supplementary file 3 — Figure S3. Trajectory analysis of penis form. [file EVL3-2-557-s003.pdf]

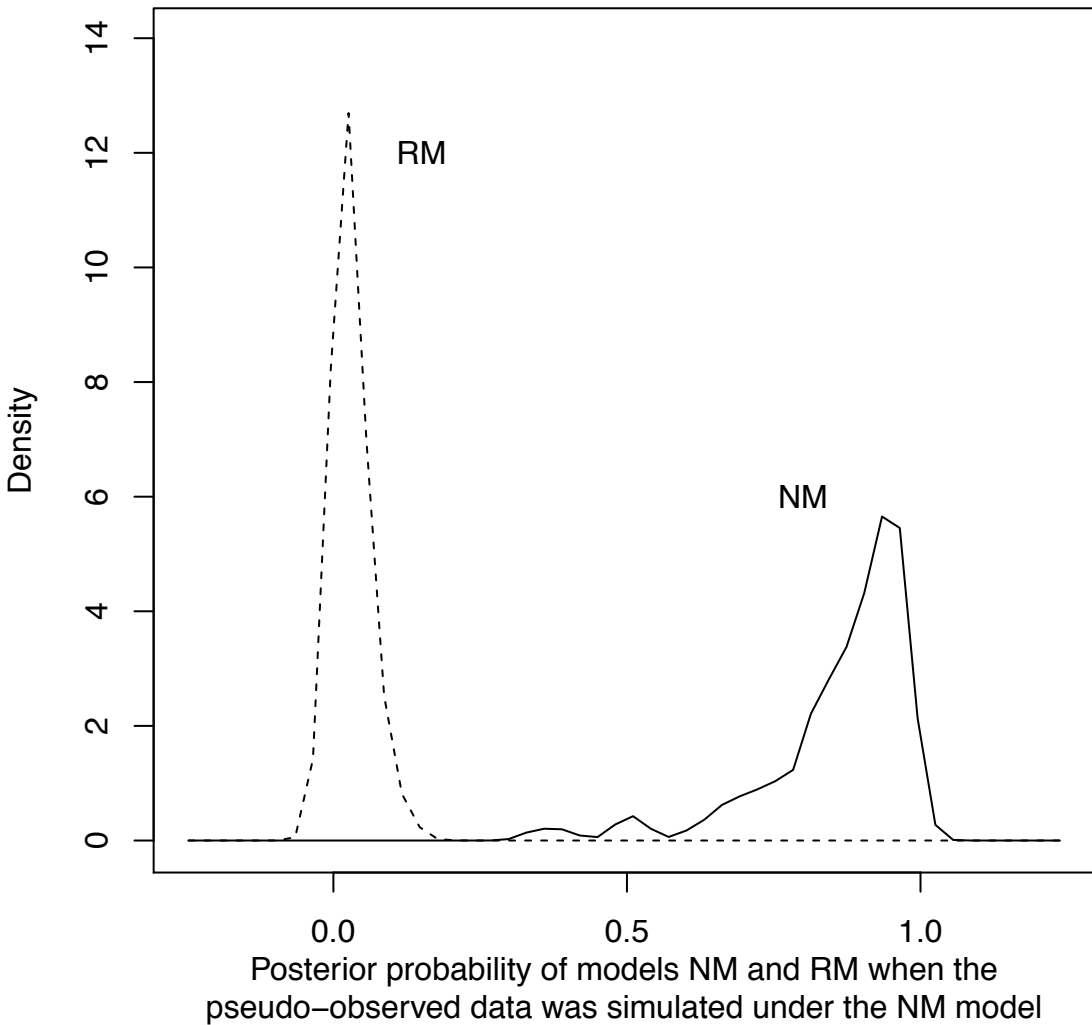

Supplement: Supplementary file 4 — Figure S4. Posterior probabilities of models NM and RM over 100 rounds of leave‐one‐out cross‐validation analysis. [file EVL3-2-557-s004.pdf]

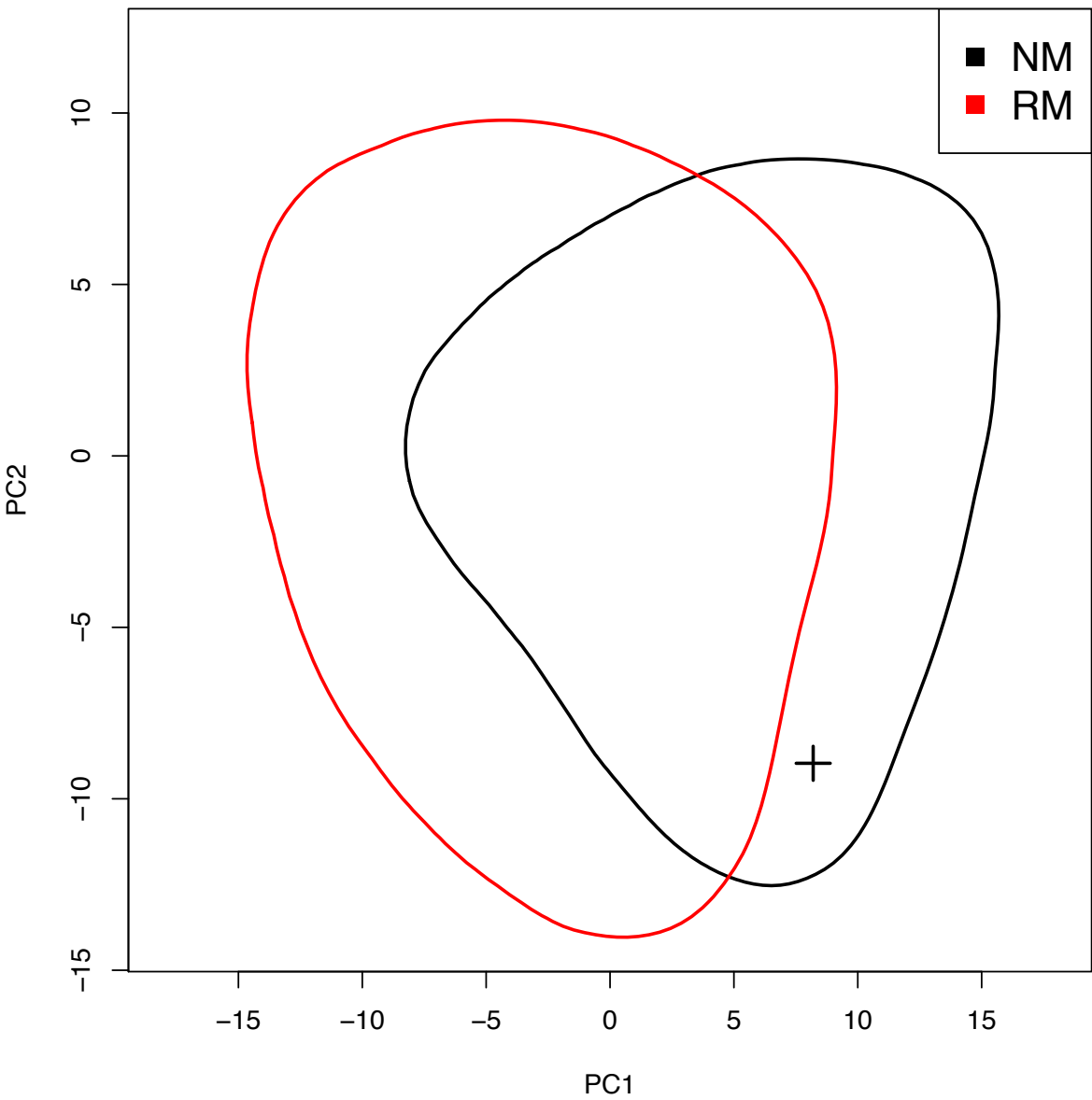

Supplement: Supplementary file 5 — Figure S5. Principal component analysis of datasets simulated under models NM and RM (1e6 datasets under each model) using the a priori simulated summary statistics. [file EVL3-2-557-s005.pdf]

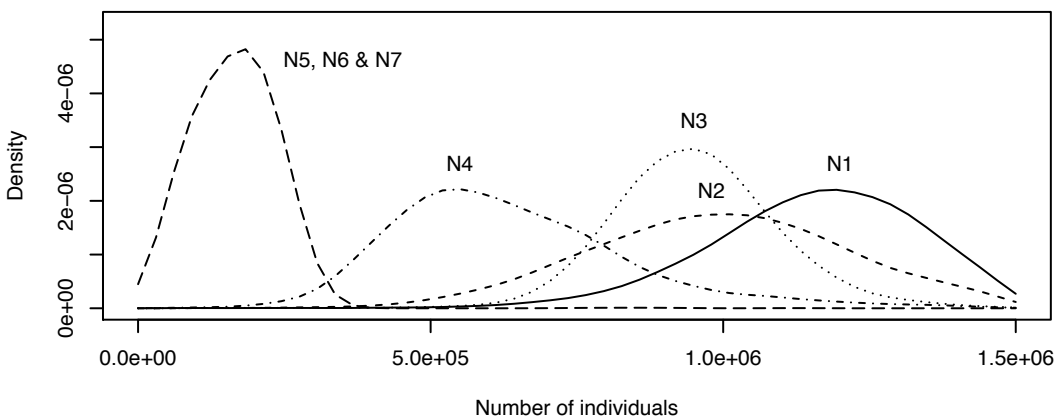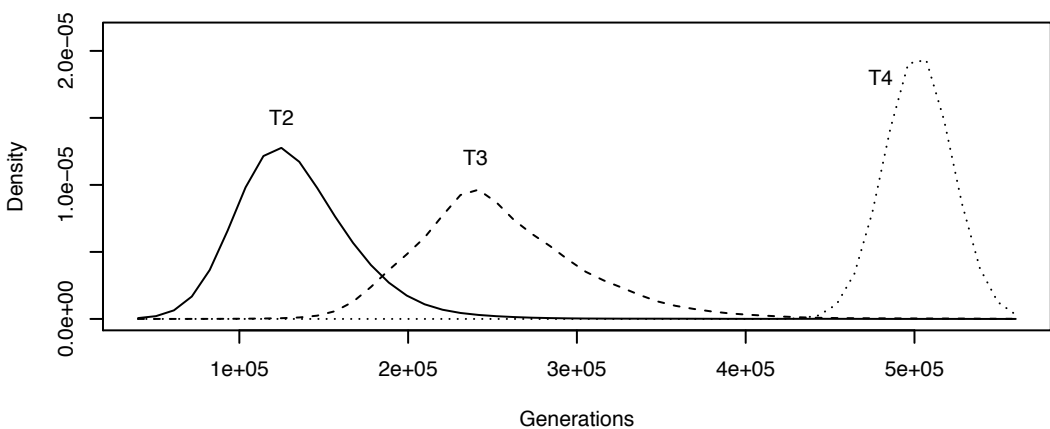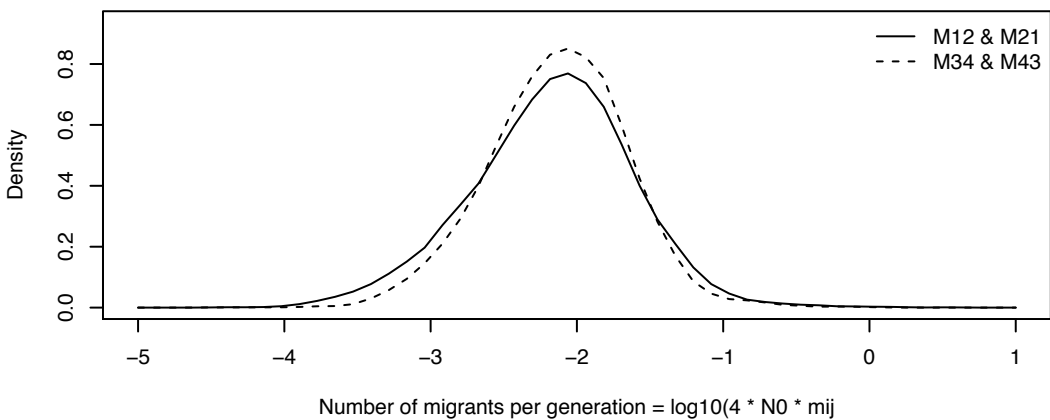

Supplement: Supplementary file 6 — Figure S6. Posterior distributions of the NM model parameters. [file EVL3-2-557-s006.pdf]
