# Supplementary material for: Are assortative mating and genital divergence driven by reinforcement?
Source: Evol Lett. 2018 Oct 16;2(6):557–66. doi: 10.1002/evl3.85 (PMC6292706; doi:10.1002/evl3.85)
Supplement: Supplementary file 2 — Figure S2. Demographic models investigated in this study. [file EVL3-2-557-s002.pdf]

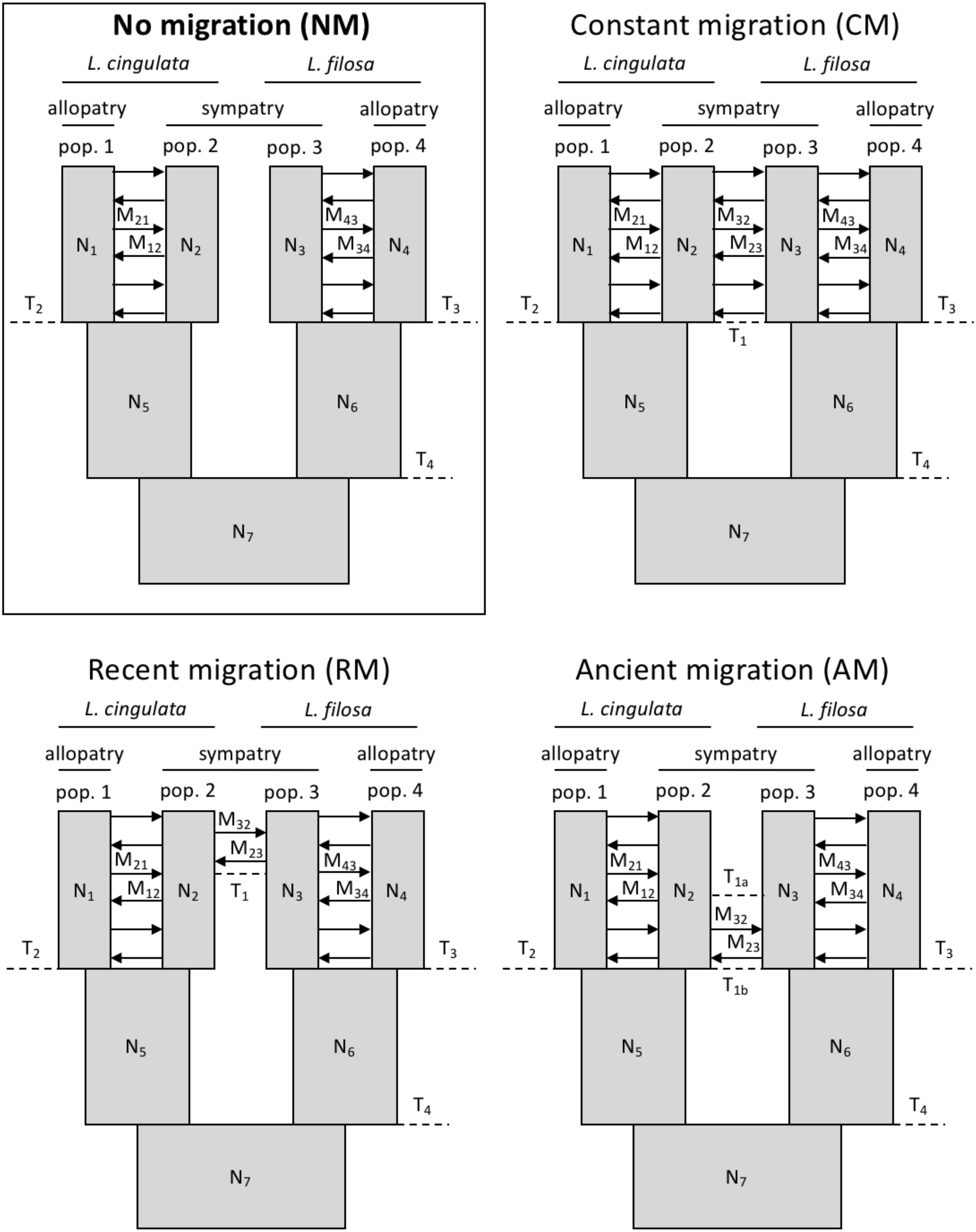
No migration (NM)
L. cingulata
L. filosa
allopatry
sympatry
allopatry
pop. 1
pop. 2
pop. 3
pop. 4
M21
M12
M43
M34
N1
N2
N3
N4
T2
T3
N5
N6
T4
N7
Constant migration (CM)
L. cingulata
L. filosa
allopatry
sympatry
allopatry
pop. 1
pop. 2
pop. 3
pop. 4
M21
M12
M32
M23
M43
M34
N1
N2
N3
N4
T2
T1
T3
N5
N6
T4
N7
Recent migration (RM)
L. cingulata
L. filosa
allopatry
sympatry
allopatry
pop. 1
pop. 2
pop. 3
pop. 4
M32
M23
M21
M12
T1
M43
M34
N1
N2
N3
N4
T2
T3
N5
N6
T4
N7
Ancient migration (AM)
L. cingulata
L. filosa
allopatry
sympatry
allopatry
pop. 1
pop. 2
pop. 3
pop. 4
M21
M12
T1a
M32
M23
T1b
M43
M34
N1
N2
N3
N4
T2
T3
N5
N6
T4
N7
