# Supplementary material for: Are assortative mating and genital divergence driven by reinforcement?
Source: Evol Lett. 2018 Oct 16;2(6):557–66. doi: 10.1002/evl3.85 (PMC6292706; doi:10.1002/evl3.85)
Supplement: Supplementary file 7 — Table S1. Collection sites of Littoraria cingulata and L. filosa. Table S2. Analysis of individual penis traits (after size correction). Table S3. Uniform prior distributions [low bound – high bound], after having ensured that priors included the posteriors. Table S4. Parameter estimation under the NM model, chosen for having received the highest posterior probability. Table S5. Primer sequences for 10 putatively diagnostic SNPs. [file EVL3-2-557-s007.pdf]

Supplementary Methods

Automated image analysis for penis measurements

An ImageJ macro (available from https://imagej.nih.gov/ij/) performed the following analysis of penis drawings. The first step of the image analysis procedure was to determine the middle line of the penis structure (Fig. S1). This line was drawn fitting a cubic spline using 8 key-points defined as follow:

1. Tip of the penis;
2. Point with equal distance from both sides of the penis and half distance between points 1 and 3;
3. As point 2 but at the widest section of the filament;
4. As point 2 but between points 3 and 5;
5. Middle point of the constriction line;
6. As point 2 but at the widest section below the filament and above the bifurcation;
7. Point of bifurcation between centre lines of penis and side branch structures;
8. Middle point of the base line.

The sequence in which the points were determined was: 5, 1, 3, 2, 4, 8, 7 and 6. Points 5 and 8 were the middle points of the hand-draw line before the scanning procedure (see above). Points 2, 3, 4, and 6 were the coordinates of the maximum of the Euclidian Distance Map (EDM) of the horizontal penis section defined by the points' relative uppermost and lowest limits (see list above). Points 1 and 7 were determined as the upper tip and the bifurcation point with the longest branches of the skeletonized filter applied to the entire penis shape, respectively. To verify that the middle line was correctly computed, we inspected by eye all images before proceeding to the measurement procedure described below.

The 11 morphometric features measured were defined as follow (Fig. S1):

A. Total penis length (from point 1 to point 8);
B. Maximum width of the section below the filament;
C. Maximum width of the penis base;
D. Filament length (from point 1 to point 5);
E. Base length (from point 5 to point 8);
F. Distance from the gland to base bifurcation (i.e., to point 7);
G. Maximum width of the side branch;
H. Maximum width of the filament;
I. Width of the constriction;
J. Maximum width of the gland (i.e., calliper diameter);
K. Width of the gland perpendicular to J.

Lengths A, D and E were measured on the middle penis line defined above. Widest sections B, C, G and H were measured as the diameter of the largest inscribed circle along selected sections of the penis' structure (Fig. S1). More precisely: B and H were defined at the points 6 and 3, respectively (see above); C was measured between points 7 and 8; F was measured between the gland attachment and point 7. The largest inscribed circle was drawn as the

circle centred in the EDM maximum coordinates and radius equal to EDM maximum of each section as defined above. The distance F was measured as the length of the line connecting: i) the middle point of the gland attachment line (hand drawn before scanning; see below), ii) the middle coordinates of G and iii) point 7. Finally, K was defined as perpendicular to J in J's middle point and thus by definition $K \leq J$. Since C could not be measured in all 224 specimens, it was excluded from downstream analysis.

Demographic inference using approximate Bayesian computation (ABC)

Simulations were carried out with the programme msnseg, a modified version of the coalescent sampler msnsam (1, 2) kindly provided by Khalid Belkhir (Institut des Sciences de l'Évolution, Montpellier). Within datasets, for each simulated locus, up to 30 attempts were allowed before observing between 1 and 4 SNPs, as in the observed data. If a number in this range was not observed after 30 attempts, then the last attempt was retained. Once a complete dataset of 29,623 loci had been simulated, it was discarded if >1% of simulated loci had a number of SNPs outside the range in the observed data. 10^6 data sets were simulated under each model.

Parameters of the models (Fig. S2) were scaled by a factor $N_0 = 10^4$. Mutation rate (μ) was $3x10^{-9}$ mutations per base per generation. We simplified models by constraining ancestral population sizes to be equal ($N_5 = N_6 = N_7$). Split times between sympatric and allopatric regions for the two species (T_2 and T_3) were not constrained relative to one another. The original ranges of priors were defined based on information available in the literature (3–5) and our field observations of density (Table S3). We used uniform distributions on a linear scale except for migration parameters, which were on a $\log_{10}$ scale in order to concentrate the sampling on relatively small values. Priors were adjusted after exploratory analysis of 10^5 simulations, in order to ensure that posteriors were fully included (Table S3).

To allow for heterogeneity in interspecific migration for different parts of the genome, we followed the approach of Roux et al. (6). Migration rates for individual loci were independently sampled from a beta distribution, drawing the parameters α and β from uniform prior distributions. Gene flow rates were then scaled between 0 and "c". In order to account for asymmetrical interspecific gene flow in sympatry, the scalar "c" was independently sampled twice from a uniform distribution for each simulated dataset. Thus, for models CM, RM, and AM, interspecific gene flow M_{23} and M_{32} were modelled as "random(Beta(α, β)) * c" for each locus.

A set of summary statistics was computed for each of the four populations and for all possible population pairs, and used for the ABC inference. For each dataset (observed or simulated), means and standard deviations were calculated across loci for the following summary statistics (using msums, a modified version of mscalc (6)): sum of pairwise differences, number of segregating sites per locus, number of singleton sites per locus, Tajima's D (7), Tajima's theta (nucleotide diversity), Watterson's theta (8), Fu and Li's D* and F* (9), Ramos-Onsins and Rozas' R2 (10), raw nucleotide divergence (Nei's Dxy, equation 12.66 (11), net nucleotide divergence (Nei's DA, equation 12.67 (11), F_{ST} (12)), and number of biallelic sites. In total, 120 summary statistics were used considering nine statistics for

four populations, four statistics for six population pairs, and two values for each statistic, i.e. mean and standard deviation. For each model, the full set of summary statistics was transformed via Partial Least Squares (13) in order to reduce its dimensionality. Transformations were performed in the R package *pls* (version 2.5-0 (14)) and the 15 PLS components that best explained the variance were retained based on visual inspection of the root mean squared error plots. The transformed statistics were then used for computing the Euclidean distance between the observed and simulated datasets for the ABC rejection step. Up to 1% (i.e. 10^4 datasets) of the transformed statistics nearest to the observed data were used for multivariate model parameter estimation via the non-linear regression correction algorithm "neural network" as implemented in the R package *abc* (version 2.1 (15)). Distributions of the posteriors were plotted using the R package *sm* (version 2.1 (16)). A symmetric credible interval of 0.9 was used for defining the lower and upper plausible limits for the estimated parameters.

Posterior model probabilities were estimated based on the untransformed summary statistics using the R package *abc* (version 2.1 (15)). Euclidean distances were computed with the "neural network" algorithm and used for choosing the 1% of simulated datasets that were closest to the observed data. The model with the highest posterior probability was chosen over the other models. In order to examine the robustness of the highest posterior model probability, an exhaustive leave-one-out cross-validation technique was used as implemented in the R package *abc*. The cross-validation ran over 100 rounds, each of which involved selecting a simulated dataset as a test dataset, while the rest of the simulated datasets were treated as training datasets. The density of the posterior model probabilities from the 100 cross-validation rounds was plotted using the R package *sm*. Here, only the two models with the highest posterior probabilities in the model selection step were included for the purpose of comparison.

In order to check the *a priori* goodness of fit of the two models with highest posterior probabilities, a PCA of the 120 summary statistics was performed for the 10^6 datasets simulated under each of these models using the R package *abc*. The first two principal components were plotted, displaying only envelopes containing 95% simulated summary statistics for each model. The observed summary statistics were also displayed in order to check whether their projection lay within the envelope of a particular model.

Putative hybrid between L. cingulata *and* L. filosa

During sampling at Broome, a putative hybrid between *L. cingulata* and *L. filosa* was identified, based on shell morphological traits (yellow colour typical of *L. filosa*; regular rounded ribs typical of *L. cingulata*). In order to test whether the individual was a hybrid we genotyped a set of SNPs with species-specific alleles. We identified SNPs with $F_{ST} = 1.0$ between species in the dataset from 113 individuals generated for testing for gene flow. For tags with sufficient sequence information, we used Primer3 (version 4.0.0 (17, 18)) for primer design. Details of primers that provided single products that could be sequenced successfully are provided in Table S5. In most tags, SNP positions were located close to the primer-binding region, increasing the risk of unreliable allele calling near the ends of sequence reads. In order to avoid this potential problem, the expected length of PCR-

amplified products was increased by binding M13-primer sequences to the PCR-primers and using them for sequencing.

Fifteen female individuals not included in the gene flow analysis were analysed with these primers. This included seven *L. cingulata* and seven *L. filosa* (all from the sympatric site Broome) in order to confirm that the 10 SNPs were diagnostic, as well as the one putative hybrid between the two species (from Broome). PCR reactions were performed in a volume of 10 µl, which included 4 µl Taq PCR Master Mix (QIAGEN), 1 µM each primer (i.e. forward and reverse), 3 µl double deionised water and 1 µl DNA (different concentrations for each sample). Amplifications were performed under a thermocycling protocol starting at 95 °C for 15 min, followed by 35 cycles at 94 °C for 30 s, 71.5 °C for 1 min, and 71 °C for 1 min, and a final elongation step at 60 °C for 30 min. The University of Sheffield Core Genomic Facility purified and cycle-sequenced PCR products with BigDye® Terminator v3.1 chemistry (Applied Biosystems™) using a pair of primers exclusively consisting of the M13 sequences. Finally, the resulting products were analysed with a 3730 DNA Analyzer (Applied Biosystems™).

Sample identity was masked throughout the genotype scoring process in order to avoid bias towards expected genotypes. Reads were aligned using the program CodonCode Aligner (CodonCode Corporation, www.codoncode.com) and SNPs were scored manually. None of the forward reads yielded good-quality sequence data; therefore, genotyping was performed using reverse reads only.

Table S1. Collection sites of *Littoraria cingulata* and *L. filosa*.

**Site**	**Site type**	**Species**	**Latitude (º)**	**Longitude (º)**
Denham (De)	Allopatric	*L. cingulata*	–25.950482	113.559362
Monkey Mia (Mo)	Allopatric	*L. cingulata*	–25.798481	113.721269
Broome (Br)	Sympatric	*L. cingulata*<br>*L. filosa*	–17.969273	122.237519
Port Smith (Po)	Sympatric	*L. cingulata*<br>*L. filosa*	–18.512117	121.804787
Darwin (Da)	Allopatric	*L. filosa*	–12.408549	130.832430
Dundee Beach (Du)	Allopatric	*L. filosa*	–12.734647	130.356864

Table S2. Analysis of individual penis traits (after size correction).

**Trait** (see Fig. S1)	**Mean (standard error)**				$\mathbf{F_{1,791}}$		
	L. cingulata in allopatry	*L. cingulata* in sympatry	*L. filosa* in sympatry	*L. filosa* in allopatry	Species	Location	Interaction
**B**	0.8603 (0.0047)	0.8034 (0.0047)	0.6749 (0.0042)	0.8103 (0.0047)	106.9***	110.9***	18.7***
**D**	2.7617 (0.0176)	2.6661 (0.0166)	3.1580 (0.0162)	3.2042 (0.0168)	193.0***	4.4	0.6
**E**	4.2579 (0.0176)	4.3537 (0.0166)	3.8617 (0.0162)	3.8155 (0.0168)	190.5***	4.8	0.3
**F**	1.2399 (0.0137)	1.5665 (0.0145)	1.7197 (0.0161)	1.5525 (0.0206)	51.2***	57.3***	5.8
**G**	1.3902 (0.0079)	1.4469 (0.0099)	1.1575 (0.0090)	1.3887 (0.0110)	67.8***	20.7***	57.4***
**H**	0.6857 (0.0046)	0.7111 (0.0040)	0.6502 (0.0043)	0.7093 (0.0063)	5.2	2.9	18.9***
**I**	0.5084 (0.0040)	0.4847 (0.0032)	0.4085 (0.0027)	0.4951 (0.0045)	44.6***	52.3***	18.6***
**J**	1.6037 (0.0096)	1.8209 (0.0129)	1.7979 (0.0139)	1.7532 (0.0181)	4.8	22.4***	9.5*
**K**	1.3410 (0.0091)	1.5408 (0.0096)	1.5211 (0.0113)	1.4590 (0.0167)	4.1	30.2***	8.4*

* P < 0.05, *** P < 0.01, after Bonferroni correction

Table S3. Uniform prior distributions [low bound – high bound], after having ensured that priors included the posteriors. Population size parameters (N) are given in number of individuals; time parameters (T) are given in generations (assuming one generation per year); migration parameters (M) are given in number of migrants per generation, i.e. $4N_0m_{ij}$, where N_0 = 1e4; NA – not applicable. Models were: NM – No migration, CM – Constant migration, RM – Recent migration, AM – Ancient migration.

	Demographic models			
Parameter	NM	CM	RM	AM
N_1	[5e4, 1.5e6]	[5e4, 1.5e6]	[5e4 – 1.5e6]	[5e4, 1.5e6]
N_2	[5e4, 1.5e6]	[5e4, 1.5e6]	[5e4 – 1.5e6]	[5e4, 1.5e6]
N_3	[5e4, 1.5e6]	[5e4, 1.5e6]	[5e4 – 1.5e6]	[5e4, 1.5e6]
N_4	[5e4, 1.5e6]	[5e4, 1.5e6]	[5e4 – 1.5e6]	[5e4, 1.5e6]
N_5, N_6 & N_7	[5e4, 1.5e6]	[5e4, 1.5e6]	[5e4 – 1.5e6]	[5e4, 1.5e6]
T_1	NA	min(T_2, T_3)	[0 – min(T_2, T_3)]	NA
T_{1a}	NA	NA	NA	[0, T_{1b}]
T_{1b}	NA	NA	NA	min(T_2, T_3)
T_2	[0, T_4]	[0, T_4]	[50 – T_4]	[50, T_4]
T_3	[0, T_4]	[0, T_4]	[50 – T_4]	[50, T_4]
T_4	[5e5, 5e6]	[5e5, 5e6]	[5e5 – 5e6]	[5e5, 5e6]
M_{12} & M_{21}	1e[-3, 1]	1e[-3, 1]	1e[-3 – 1]	1e[-3, 1]
M_{34} & M_{43}	1e[-3, 1]	1e[-3, 1]	1e[-3 – 1]	1e[-3, 1]
alpha	NA	[0 – 20]	[0, 20]	[0, 20]
beta	NA	[0 – 500]	[0, 500]	[0, 500]
c_{23}	NA	[0 – 15]	[0, 15]	[0, 15]
c_{32}	NA	[0 – 15]	[0, 15]	[0, 15]

Table S4. Parameter estimation under the NM model, chosen for having received the highest posterior probability. Population size parameters (N) are given in number of individuals; time parameters (T) are given in generations (assuming one generation per year), and migration parameters (M) are given in number of migrants per generation, i.e. $4N_0m_{ij}$, where N_0 = 1e4. Posterior distributions are given in Fig. S6.

				Credible interval 90%	
Parameter	Median	Mean	Mode	Lower	Upper
N_1	1.17e6	1.16e6	1.18e6	0.85e6	1.42e6
N_2	1.00e6	0.99e6	1.02e6	0.63e6	1.36e6
N_3	0.95e6	0.96e6	0.96e6	0.75e6	1.18e6
N_4	0.61e6	0.64e6	0.53e6	0.37e6	1.04e6
N_5, N_6 & N_7	0.16e6	0.16e6	0.18e6	0.06e6	0.25e6
T_2	1.2e5	1.3e5a	1.1e5	1.0e5	1.8e5
T_3	2.4e5	2.5e5	2.3e5	2.0e5	3.3e5
T_4	5.0e5	5.0e5	5.0e5	5.0e5	5.0e5
M_{12} & M_{21}	7.1e-3	17.4e-3	3.6e-3	0.7e-3	49.4e-3
M_{34} & M_{43}	7.5e-3	13.8e-3	3.7e-3	1.1e-3	38.8e-3

Table S5. Primer sequences for 10 putatively diagnostic SNPs. In primer names, numbers indicate tag number in the sequencing data set, and letters indicate direction (i.e. forward or reverse).

Primer name	Sequence 5' to 3'
458736_f	CACGACGTTGTAAAACGACCAGTCTCCAAGCCATGCAG
458736_r	CAGGAAACAGCTATGACCTGCCCAACTCGTCCTTCAAG
458866_f	CACGACGTTGTAAAACGACAGAGAAGTTGTCCACAGCCA
458866_r	CAGGAAACAGCTATGACCGGAAAGCCACCATGTAGATATTGA
459573_f	CACGACGTTGTAAAACGACTGACGTCATGGTGCTGGT
459573_r	CAGGAAACAGCTATGACCCGCTGGACATGTCACGTG
459591_f	CACGACGTTGTAAAACGACACTCTCTCTCCATCTCCTCCA
459591_r	CAGGAAACAGCTATGACCGGTGTGGACAGGTTCACAAA
460354_f	CACGACGTTGTAAAACGACCTGCCTGTTCATGCTGTGG
460354_r	CAGGAAACAGCTATGACCGGCCCCGTACAGCTATGATA
461412_f	CACGACGTTGTAAAACGACGCTGTTTCATATTTCATATCCACACC
461412_r	CAGGAAACAGCTATGACCCGTTTCAGGCTGGCACTG
638118_f	CACGACGTTGTAAAACGACTTTTCAAGGACCTCAAAACAGAC
638118_r	CAGGAAACAGCTATGACCCTTGTCTTCATGGCAGGCTC
724358_f	CACGACGTTGTAAAACGACCATAATGTCTGTATCGCCCCG
724358_r	CAGGAAACAGCTATGACCGGCGGTCCTCAGGCTGTC
726135_f	CACGACGTTGTAAAACGACTGTGTAGGGTGTGGATGAGAC
726135_r	CAGGAAACAGCTATGACCTGCTGATGGTTCTTCTGTGC
759829_f	CACGACGTTGTAAAACGACAGCAGAAAATCACACAACCGT
759829_r	CAGGAAACAGCTATGACCTTGAACGAAAGGCAGACGAC

Fig. S1. Feature extraction from penis drawings (see *Supplementary Methods* for details).

Fig. S2. Demographic models investigated in this study. The framed model (NM) was the model with the highest posterior probability. Bottom of diagrams represents the past, while top of diagrams represents the present time. $N_{1,2,3,4}$ – current sizes of populations 1, 2, 3, and 4, respectively; $N_{5,6}$ – ancestral population sizes of *L. cingulata* and *L. filosa*, respectively; N_7 – common ancestral population size; T_1 and T_{1b} – time since beginning of interspecific gene flow; T_{1a} – time since cessation of interspecific gene flow; $T_{2,3,4}$ – time since the *L. cingulata*, *L. filosa*, and most ancient split, respectively; M_{ij} – migration rate into population *i* from population *j*. For the CM model, T_1=min(T_2, T_3).

Fig. S3. Trajectory analysis of penis form. Blue is *L. cingulata*, black is *L. filosa* (centroids). Lines are the between-species trajectories for allopatric and sympatric populations, projected onto the first two principal components. They differ significantly in orientation, but not in magnitude.

Fig. S4. Posterior probabilities of models NM and RM over 100 rounds of leave-one-out cross-validation analysis.

Fig. S5. Principal component analysis of datasets simulated under models NM and RM (1e6 datasets under each model) using the *a priori* simulated summary statistics. Lines represent envelopes containing 95% simulations for each model. The cross indicates the projection of the observed summary statistics.

Fig. S6. Posterior distributions of the NM model parameters. Population size parameters (N) are given in number of individuals; time parameters (T) are given in generations (assuming one generation per year), and migration parameters (M) are given in log_{10} number of migrants per generation, i.e. $log_{10}(4N_0m_{ij})$, where N_0 = 1e4.

1. Hudson RR (2002) Generating samples under a Wright–Fisher neutral model of genetic variation. *Bioinformatics* 18:337–338.
2. Ross-Ibarra J, et al. (2008) Patterns of polymorphism and demographic history in natural populations of *Arabidopsis lyrata*. *PLoS ONE*, 3:e2411.
3. Reid DG (1986) The Littorinid molluscs of mangrove forests in the Indo-Pacific region: the genus *Littoraria.* British Museum (Natural History), London.
4. Turney CSM, et al. (2006) Integration of ice-core, marine and terrestrial records for the Australian Last Glacial Maximum and Termination: a contribution from the OZ INTIMATE group. *J Quat Sci* 21:751–761.
5. Reid DG, Dyal P, Williams ST (2012) A global molecular phylogeny of 147 periwinkle species (Gastropoda, Littorininae). *Zoo Scrip* 41:125–136.

6. Roux C, Tsagkogeorga G, Bierne N, Galtier N (2013) Crossing the species barrier: genomic hotspots of introgression between two highly divergent *Ciona intestinalis* species. *Mol Biol Evol* 30:1574–1587.
7. Tajima F (1989) Statistical method for testing the neutral mutation hypothesis by DNA polymorphism. *Genetics* 123:585–595.
8. Watterson GA (1975) On the number of segregating sites in genetical models without recombination. *Theor Pop Biol* 7:256–276.
9. Fu Y-X, Li W-H (1993) Statistical tests of neutrality of mutations. *Genetics*, 133:693–709.
10. Ramos-Onsins SE, Rozas J (2002) Statistical properties of new neutrality tests against population growth. *Mol Biol Evol* 19:2092–2100.
11. Nei M, Kumar S (2000) Molecular evolution and phylogenetics. Oxford University Press, Oxford.
12. Hudson RR, Slatkin M, Maddison WP (1992) Estimation of levels of gene flow from DNA sequence data. *Genetics* 132:583–589.
13. Boulesteix A-L, Strimmer K (2006) Partial least squares: a versatile tool for the analysis of high-dimensional genomic data. *Brief Bioinfo* 8:32–44.
14. Mevik B-H, Wehrens R (2007) The pls package: Principle Component and Partial Least Squares Regression in R. *J Stat Soft* 18:1–23.
15. Csilléry K, François O, Blum MGB (2012) abc: an R package for approximate Bayesian computation (ABC). *Meth Ecol Evol* 3:475–479.
16. Bowman AW, Azzalini A (2003) Computational aspects of nonparametric smoothing with illustrations from the sm library. *Comput Stat Data Anal* 42:545–560.
17. Koressaar T, Remm M (2007) Enhancements and modifications of primer design program Primer3. *Bioinformatics* 23:1289–1291.
18. Untergasser A et al. (2012) Primer3—new capabilities and interfaces. *Nucl Aci Res* 40:e115.
